# Physicochemical properties, mechanism of action of lycopene and its application in poultry and ruminant production

**DOI:** 10.3389/fvets.2024.1364589

**Published:** 2024-03-18

**Authors:** Yong Long, Siwaporn Paengkoum, Shengyong Lu, Xinran Niu, Sorasak Thongpea, Nittaya Taethaisong, Yong Han, Pramote Paengkoum

**Affiliations:** ^1^School of Animal Technology and Innovation, Institute of Agricultural Technology, Suranaree University of Technology, Nakhon Ratchasima, Thailand; ^2^Program in Agriculture, Faculty of Science and Technology, Nakhon Ratchasima Rajabhat University, Nakhon Ratchasima, Thailand; ^3^Guizhou University of Traditional Chinese Medicine, Guiyang, China

**Keywords:** antioxidant, anti-cancer, additive, lipid-lowering, lycopene

## Abstract

Lycopene is a kind of natural carotenoid that could achieve antioxidant, anti-cancer, lipid-lowering and immune-improving effects by up-regulating or down-regulating genes related to antioxidant, anti-cancer, lipid-lowering and immunity. Furthermore, lycopene is natural, pollution-free, and has no toxic side effects. The application of lycopene in animal production has shown that it could improve livestock production performance, slaughter performance, immunity, antioxidant capacity, intestinal health, and meat quality. Therefore, lycopene as a new type of feed additive, has broader application prospects in many antibiotic-forbidden environments. This article serves as a reference for the use of lycopene as a health feed additive in animal production by going over its physical and chemical characteristics, antioxidant, lipid-lowering, anti-cancer, and application in animal production.

## 1 Introduction

**Figure 1:**
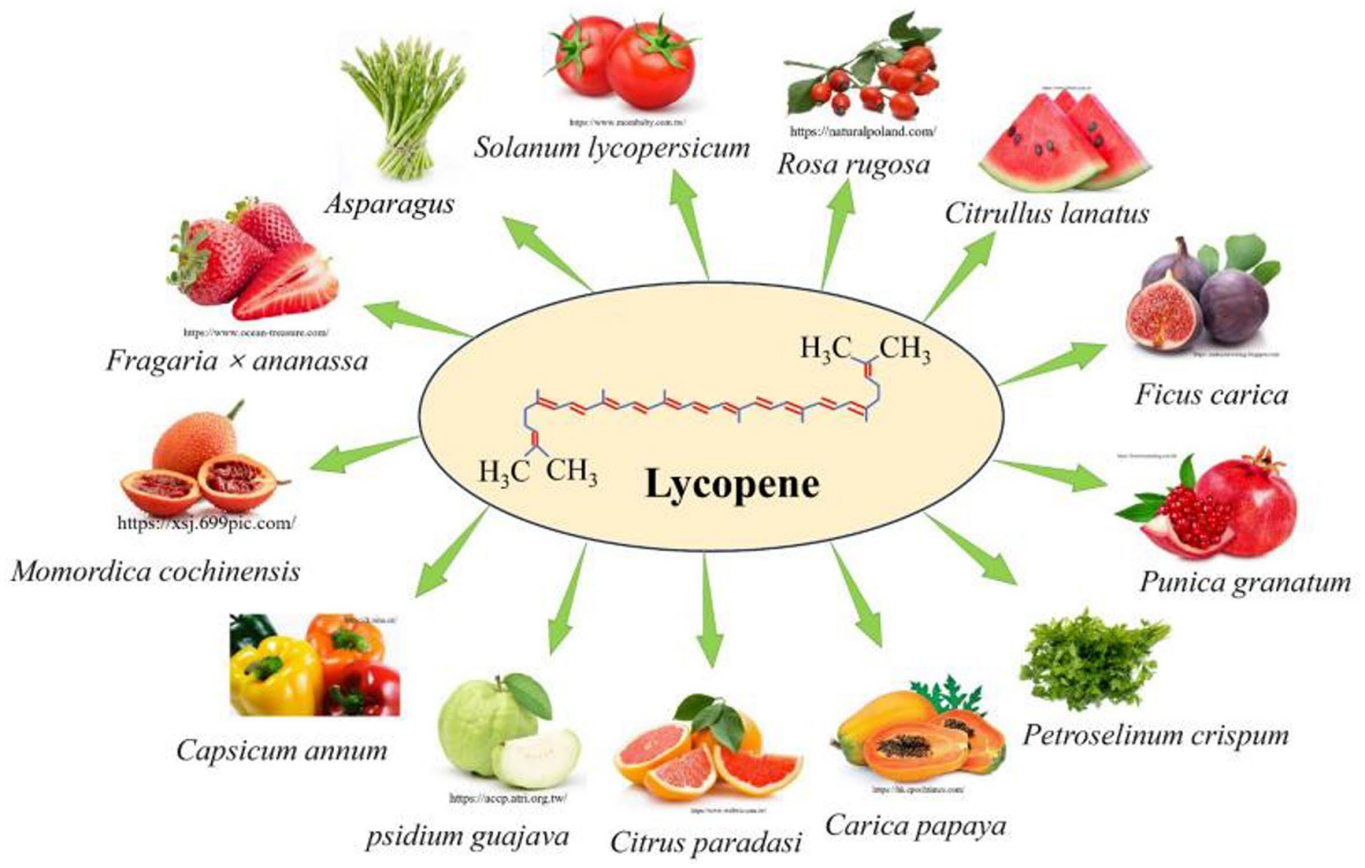
Sources of lycopene in food (1, 2).

Lycopene was an acyclic isomer of *β-carotene* and a natural, non-polluting, non-toxic pigment, which found primarily in red or orange fruits and vegetables, such as *Solanum lycopersicum*, *Carica papaya*, *Psidium guajava*, *Citrullus lanatus*, and *Punica granatum* (1) (Figure 1). Furthermore, lycopene also found in certain non-red and orange foods, such as *Asparagus* and *Petroselinum crispum* (2) and marine halophilic archaea can also produce lycopene (3). However, the human body cannot synthesize lycopene by itself, and 85% of the lycopene needed by the human body mostly comes from tomatoes or tomato-based products (4). The content of lycopene in fresh tomatoes and tomato products is shown in Table 1. The absorption efficiency of the human body for lycopene was 10–30%, and excessive intake will be excreted from the body (6–8). The current methods for extracting lycopene mainly include classic organic solvent extraction, pulsed electric field, enzyme-assisted extraction, supercritical fluid extraction, ultrasonic-assisted extraction, microwave-assisted extraction, and Water-Induced hydro colloidal complexation (9). Lycopene had anti-cancer (10), antioxidant (11), anti-inflammatory (12), regulating body metabolism (13, 14), and immunity (15), and so on. Currently, reports about lycopene are gradually increasing. Previous research has shown that lycopene improved animal production performance (16), maintained intestinal health (17), ameliorated meat quality (18), ameliorated immunity (19) and regulated body metabolism (20), and has good application prospects.

**Table 1:** Lycopene content in common fresh tomatoes and tomato products (5).

Items	Lycopene (wet weight), mg/100 g	
	Range	Mean ± SD^a^
Tomatoes (fresh)	1.82–11.19	5.26 ± 2.40
Tomato puree	5.56–16.94	10.21 ± 3.20
Ketchup (hot)	5.42–52.20	24.27 ± 16.70
Ketchup (mild)	4.84–41.39	14.25 ± 11.93
Ketchup (plain)	5.08–24.96	8.10 ± 5.79
Tomato paste (double cone)	3.80–49.46	25.22 ± 14.87
Tomato juice	6.93–42.74	20.10 ± 13.83
Whole canned tomatoes	5.87–42.14	16.98 ± 14.39

a “Mean ± SD” means Mean ± standard deviation.

The importance of lycopene has been a research hotspot in recent decades. This article will focus on a more comprehensive review of the physiological functions, mechanisms of action, and application of lycopene in animal production. Therefore, this latest review will ultimately provide more useful information and the latest research perspectives for animal husbandry researchers for animal production, to provide the latest reference for the functional utilization of lycopene as a feed additive.

## 2 Physicochemical properties and safety of lycopene

### 2.1 Physicochemical properties of lycopene

**Figure 2:**
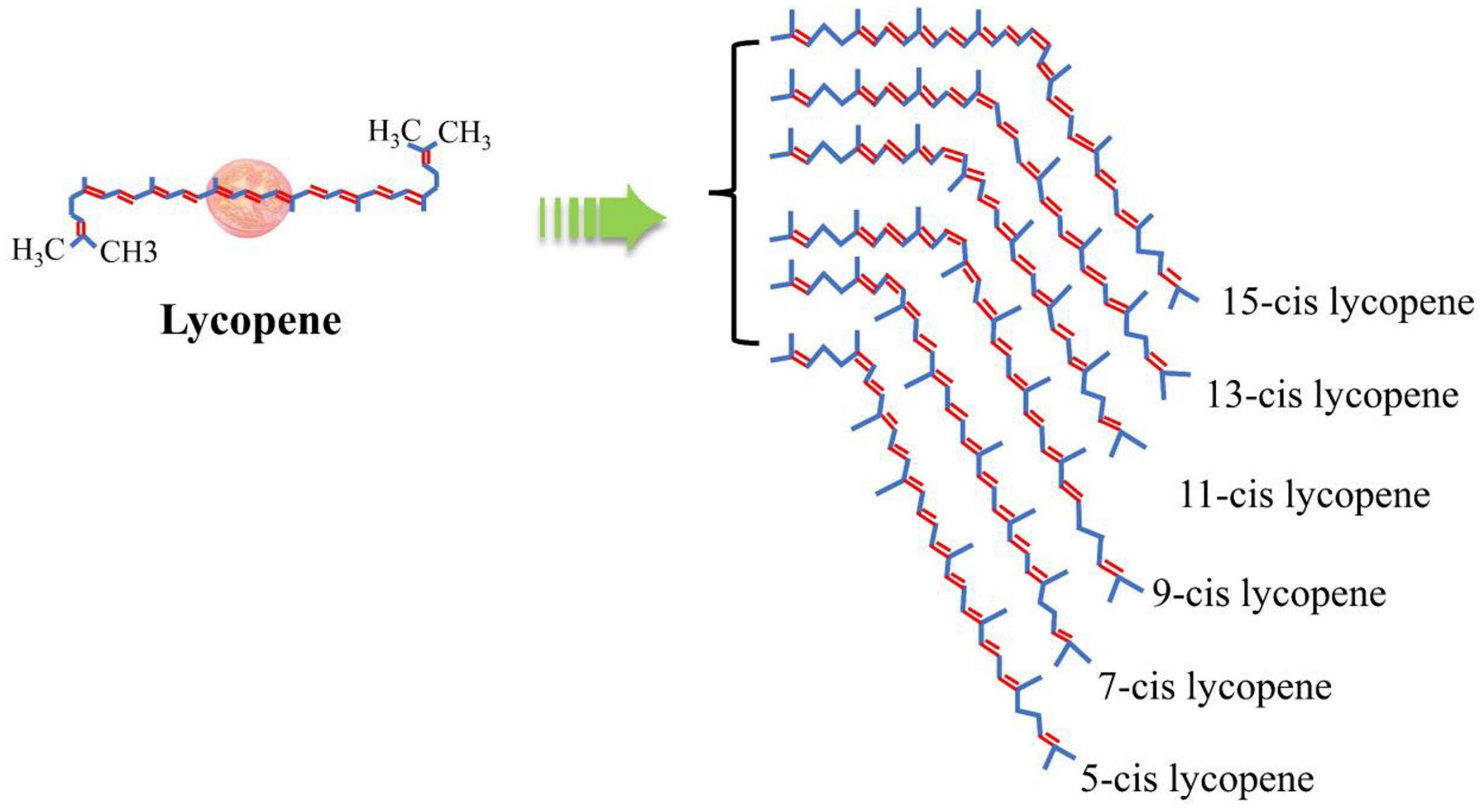
Chemical structure of lycopene (21).

Lycopene was an isoprenoid unsaturated olefin compound and a fat-soluble carotenoid. Lycopene consists of 40 carbon chains, including 11 conjugated double bonds and 2 non-conjugated double bonds (Figure 2). Its molecular formula is C_40_H_56_, its relative molecular mass is 536.85, and its melting point ranges from 172 to 175°C. Lycopene has obvious lipophilicity and is easily soluble in organic solvents, such as Hexane (C_6_H_14_), chloroform (CHCl_3_), carbon disulfide (CS_2_), petroleum ether, acetone (C_3_H_6_O) and benzene (C_6_H_6_), and so on. However, Lycopene is insoluble in water, and methanol, ethanol, and is sensitive to light, acid, catalysts, high temperatures, and metal ions (22). In nature, lycopene has a high degree of unsaturation and mainly appears in the form of the all-trans isomer, which easily oxidatively degrades and undergoes an isomerization reaction under the influence of light, temperature, or chemical reactions, converting from all-trans isomers to mono-cis or poly-cis isomers (23). The most stable form of lycopene among all isomers is said to be 5-cis, which is followed by all-trans, 9-cis, 13-cis, 15-cis, 7-cis, and 11-cis (24). Consequently, all-trans isomers comprise 90% of the lycopene found in tomatoes, whereas most of the processed tomato products exist in the form of the cis isomer.

### 2.2 Safety of lycopene

Lycopene is harmless to animals and humans and has certain benefits, which whether in the form of preparation or crystallization, was not genotoxic under stable conditions (25), Lycopene were not toxic to rabbit lymphocytes and did not produce any mutagenic activity (26). Similar results have been obtained in subchronic and chronic safety studies in mice (27). Additionally, it was concluded by Rao et al. (7) that healthy individuals who consume 5 to 75 mg of lycopene daily will not experience any negative effects. At the same time, Kong et al. (28) indicated that a daily intake of 3 g/kg of lycopene from food will not harm the human body. Therefore, lycopene is considered a safe Non-toxic substance.

## 3 Physiological functions and mechanisms of action of lycopene

### 3.1 Antioxidant function

Under normal physiological conditions, the generation and elimination of free radicals were always in a state of dynamic balance. When oxidative stress occurs, this dynamic balance will be broken, and fat, protein, and DNA in the body will be damaged, leading to a variety of diseases (29). The advancement of animal husbandry is gravely threatened by oxidative stress, which brings huge economic losses to the industry (30). Antibiotics were once useful antioxidant medications that might enhance animal immunity, growth performance, and disease prevention. However, animal production, human health, and environmental sustainability are threatened by antibiotic resistance and residues (31). Since the European Union (EU) banned the use of antibiotics in animal feed in 2006, and researchers have been trying to find plant-based feed supplements as safe antibiotic alternatives (32). In July 2020, China announced that livestock farming had officially entered a new era of banning the use of antibiotics in feed (33). Therefore, developing natural, healthy, and safe antibiotic alternatives for animal husbandry is one of the hot spots in modern feed research.

Relevant reports indicated that lycopene due to its polyunsaturated structure, not only directly scavenged free radicals, but also indirectly scavenged free radicals by regulating the enzymatic antioxidant defense system and enzymatic oxidative damage system (34). Therefore, lycopene protects proteins, DNA, and lipids in the body from oxidative damage, thereby reducing oxidative stress in the body and maintaining animal health.

#### 3.1.1 Directly scavenge free radicals

The unique 11 conjugated double bonds in lycopene are highly reactive towards oxygen and free radicals (35). Furthermore, in different carotenoids, lycopene has an antioxidant capacity second only to astaxanthin and is an important inhibitor of reactive oxygen species (ROS). Its effectiveness in scavenging singlet oxygen is 100 times greater than vitamin E, 10 times greater than that of *α-tocopherol* , and double that of *β-carotene* (36, 37). Lycopene scavenged hydroxyl radicals through an addition reaction (38) and could also react with peroxynitrite, thus effectively functioning as a nitrite scavenger (39, 40). Moreover, lycopene also has the function of scavenging sulfuryl, nitrogen dioxide, and sulfuryl free radicals (41). Whether under polar or non-polar conditions, lycopene has a higher rate of scavenging hydrogen peroxide (H_2_O_2_) free radicals than β-carotene (42).

#### 3.1.2 Lycopene antioxidant system activity

**Figure 3:**
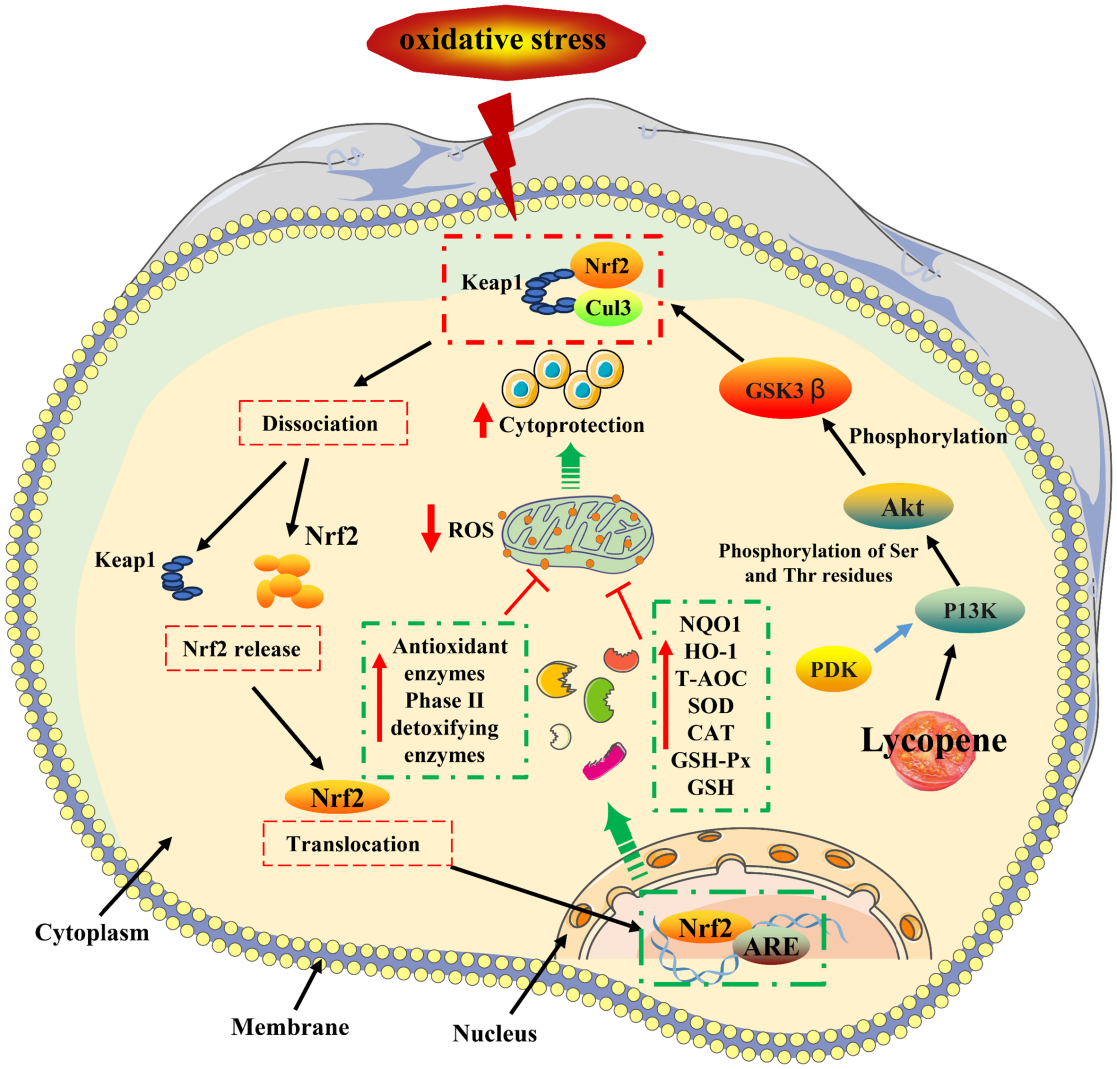
Lycopene antioxidant mechanism (10, 43). HNF1-α, Hepatocyte nuclear factor1-α; LDL-R, Hepatic LDL receptors.

The antioxidant mechanism of lycopene is shown in Figure 3. Lycopene indirectly acts on free radicals by regulating the enzymatic antioxidant defense system and enzymatic oxidative damage system, protecting the body from oxidative damage (34, 44). Shen et al. (45) have shown that lycopene promoted mouse cardiac glutathione peroxidase (GSH-Px) activity and increased cardiac glutathione (GSH) levels; at the same time, cardiac myeloperoxidase (MPO), H_2_O_2_ levels and glutathione S-transferase (GST) activity shown a decreasing trend. Therefore, their experiment proved that lycopene inhibiting di (2-ethylhexyl) phthalate-induced oxidative stress response. On the other hand, lycopene promoted the activities of GST and catalase (CAT) in the liver of ducklings and increased the total antioxidant capacity (T-AOC) of the liver, which reduced the liver malondialdehyde (MDA) content and the residual aflatoxin in the liver of ducklings obviously of the body (46). Wen et al. (47) indicated that the incorporation of 200 mg/kg lycopene in diets of swine diets increased antioxidant indices superoxide dismutase (SOD), T-AOC, GSH-Px, and CAT in serum, and reduced the levels of MDA. Relevant records have shown that lycopene increased the levels of peroxisome proliferator-activated receptor γ (*PPAR-γ*) and typical antioxidant biomarkers in rats fed a high-fat diet (48). Similarly, Li et al. (49) obtained similar results in a study on a mouse model of ulcerative colitis induced by dextran sulfate sodium. Moreover, lycopene combined with metformin increased the activity of the antioxidant enzyme paraoxonase-1 ( *PON-1* ), enhanced the endogenous oxidative defense ability of obese mice, and protected the health of the kidneys and liver (50). A previous study by Aboubakr et al. (51) indicated that lycopene effectively reduced cardiac antioxidant damage and apoptosis caused by methotrexate. Lee et al. (52) indicated that lycopene also reduced reactive ROS production by inhibiting nicotinamide adenine dinucleotide phosphate oxidase ( *NOX* ) activity, which in turn ameliorates oxidative damage to acinous cell induced by ethanol and the fatty palmitoleic acid.

**Figure 4:**
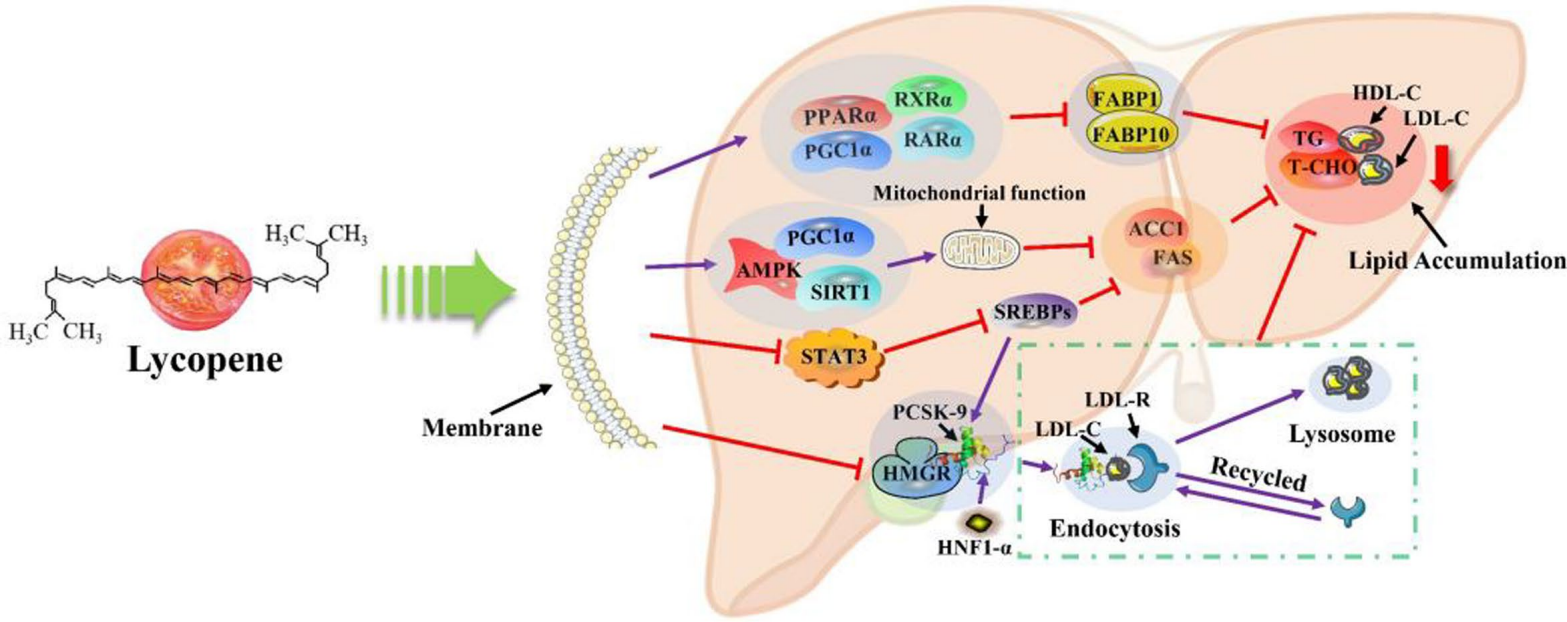
Lycopene lipid-lowering mechanism (13, 14, 53–57).

The antioxidant mechanism of lycopene was shown in Figure 4. Nuclear factor erythroid 2-related factor 2 ( *Nrf2* ) is an important transcription factor that affects the activities of CAT, SOD, GST, and GSH-Px in the oxidative system, (58, 59). Normally, the cytoplasmic kelch-like ECH-associated protein 1 (*Keap1*) is bound by the Neh2 (Nrf2-ECH homology) domain at the end of Nrf2, negatively regulating *Nrf2* and usually preventing *Nrf2* from translocating to the nucleus (60, 61). In responding to oxidative stress, the binding of *Keap1* and *Nrf2* will be rapidly dissociated, prompting *Nrf2* to enter the nucleus and bind to the antioxidant response element (ARE) sequence in the nucleus (62). Consequently, this process transcriptionally upregulates the expression of ARE and *Nrf2*, stimulating the production of phase II detoxifying antioxidant enzymes and upregulating the expression of antioxidant-related stress genes (63). Keap1/Nrf2/ARE pathway regulates NAD (P)H: quinone receptor oxidoreductase 1 ( *NQO1* ) and heme oxidase 1 ( *HO-1* ), induces the expression of various antioxidant protection genes, reduces the damage of ROS to cells, and effectively reduces the occurrence of liver diseases caused by oxidative stress (64, 65). Relevant research has shown that lycopene could act on the Nrf2/HO-1 and protein kinase B(Akt)/Nrf2 signaling pathway, induce *Nrf2* gene transcription, upregulate the mRNA expression levels of *NQO1* and *HO-1*, improve the activities of SOD, CAT, and GSH-Px, and reduce the accumulation of ROS and MDA oxidative stress end-products (66–70). Furthermore, it has been documented that lycopene inhibited β-secretase ( *BACE* ) activity by triggering the PI3K/Akt/Nrf2 signaling pathway, which then attenuates the damage of oxidative stress and apoptosis in M146L cells (43).

In conclusion, there are two basic ways that lycopene might increase the body’s antioxidant capacity. On the one hand, it can directly act on free radicals to achieve the scavenging effect. On the other hand, it can improve the mRNA expression level and activity of related antioxidant enzymes by activating the *Nrf2* antioxidant signaling pathway to achieve the purpose of scavenging free radicals and maintaining the oxidation balance of cells or the body.

### 3.2 Lycopene’s lipid-lowering function

The lipid-lowering mechanism of lycopene was shown in Figure 4. Lycopene could inhibit lipid synthesis in the body through multiple pathways of action, and at the same time accelerate the rate of lipid transport and mobilization efficiency, thereby achieving lipid-lowering effects. Peroxisome proliferator-activated receptor α ( *PPARα* ), as the most important factor in the lipid-lowering mechanism of lycopene, plays a decisive role in the lipid-lowering function of lycopene. Relevant research has shown that *PPARα* could activate β-oxidation-related genes, thereby increasing the expression of mRNA of related genes, and promote the uptake of lipid mitochondria in body cells, accelerate lipolysis, and then play an important role in lipid metabolism in the body. *PPARα* not only orchestrates the coordination of lipoprotein metabolism but also directly or indirectly controls the lipogenesis pathway in the liver (71, 72). In another study, lycopene was found to possess another important mechanism of lipid metabolism. Lycopene increased the gene expression of *PPARγ* coactivator 1α ( *PGC1α* ), *PPARα* , retinoic X receptor α ( *RXRα* ) and retinoic acid receptor α ( *RARα* ) in the liver of breeding hens, which downregulated the expression of liver fatty acid binding protein 1 ( *FABP1* ), liver fatty acid binding protein 10 ( *FABP10* ) and jejunum fatty acid transporter 4 ( *FATP4* ) genes. Moreover, lycopene also upregulated the expression of *PPARγ* , *RXRγ* , *RXRα* , and duodenal *RXRα* genes in the jejunum. Therefore, lycopene accelerates the fat metabolism rate in the body and reduces fat deposition through this pathway (55). Lycopene could activate the AMP-activated protein kinase/ Sirtuin 1/peroxisome proliferator-activated receptor-γ-coactivator 1α (AMPK/SIRT1/PGC1α) or inhibit signal transducer and activator of transcription 3 (STAT3) biosynthetic signaling pathway. and then lycopene upregulated the mRNA expression levels of SIRT1 and inhibited the mRNA expression levels of sterol regulatory element binding proteins (SREBPs), fatty acid synthase ( *FAS* ), and acetyl-CoA carboxylase 1 ( *ACC1* ) genes, thereby inhibiting the synthesis of lipids, and ultimately reducing the levels of total cholesterol (T-CHO), triglycerides (TG), and low-density lipoprotein (LDL-C) in the serum of mouse livers and broiler chickens (13, 14, 56). Moreover, lycopene reduced lipid synthesis and prevented mitochondrial dysfunction induced by palmitate, thereby reducing the risk of nonalcoholic fatty liver disease (NAFLD) in mice (13, 14, 56, 57). In addition, it was concluded by Huang et al. (73) and Albrahim et al. (48) that lycopene could improve liver damage caused by a high-fat diet in mice. Moreover, Lu et al. (74) also reported that lycopene reduced TG in HepG2 cells caused by oleic acid and palmitoleic acid. Alvi et al. (53) demonstrated that lycopene inhibits the activity of HMG-CoA reductase (HMGR) and proprotein convertase subtilisin / kexin type 9 (PCSK-9) transcription. Therefore, lycopene reduced the synthesis of T-CHO and the endocytosis of LDL-C by inhibiting the activity of HMGR and LDL-C receptors, thus achieving the purpose of lipid-lowering (54). Moreover, lycopene enhanced the activity of AMP-activated protein kinase ( *AMPK-P* ) and downregulating ATP citrate-raising lyase ( *ACLY* ). Ultimately, the levels of MDA, *HMG-CoA reductase* , *FAS* , *ACLY* , and TNF-α were reduced and the levels of *AMPK-P* and GSH were increased in diabetic hyperlipidemia rats (75).

In conclusion, through a review of many existing studies, it was found that there are four main pathways for the lipid-lowering mechanism of lycopene. Lycopene activates the *PPARα* and AMPK/SIRT1/PGC1α pathways, thereby downregulating the expressions of *FABP1* , *FABP10* , *ACC1* and *FAS* . In addition, lycopene could also downregulate the expression of *ACC1* and *FAS* and LDL-C endocytosis by inhibiting the *STAT3* biosynthetic signaling pathway and the HMGR/PCSK-9 transcriptional activity pathway, ultimately achieving a lipid-lowering effect.

### 3.3 Lycopene anti-cancer function

**Figure 5:**
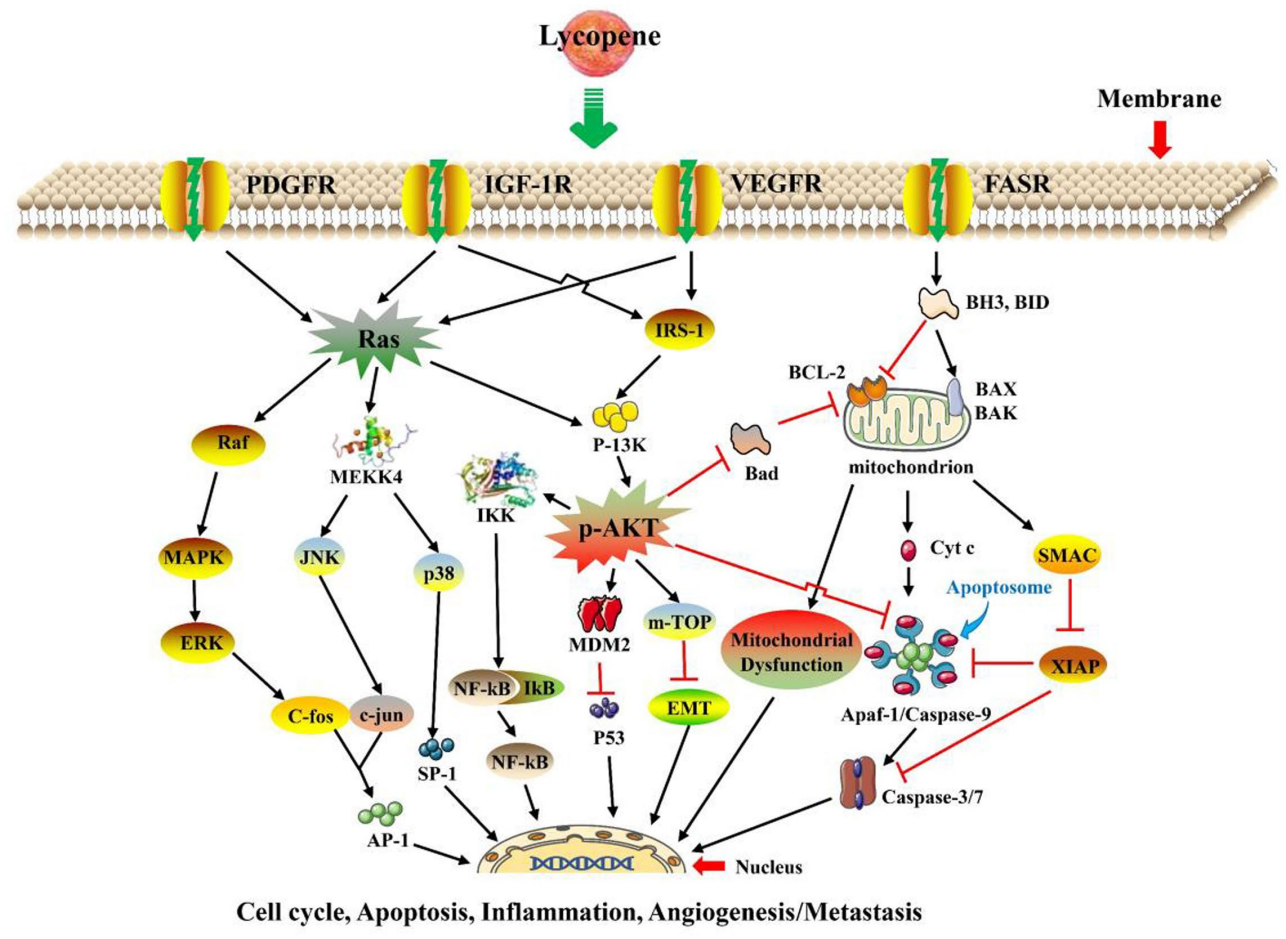
The main anti-cancer mechanism of lycopene (14, 46, 48, 76–79). MAO-A, Monoamine oxidase-A; ADA, Adenosine deaminase; NTPDase, Nucleotide triphosphatase; AchE, Acetylcholine esterase.

The anti-cancer mechanism of lycopene was shown in Figure 5. There were *in vitro* and *in vivo* research has shown that lycopene has an important anticancer effect, which inhibited the proliferation of cancer cells through antioxidant activity, regulation of anti-inflammation, growth factor signaling, apoptosis induction, and cell arrest, such as anterior prostate cancer, cervical cancer, breast cancer, melanoma, ovarian cancer, oral cancer, hepatocellular carcinoma, lung cancer, and other cancer cells (13, 14, 80–88). The content of Serum lycopene and lycopene intake was inversely proportional to the probability of cancer (10). Inducing cancer cell apoptosis is one of the most important anti-cancer mechanisms of lycopene. The main pathway of apoptotic decomposition of cells is the activation of BH3-only protein (BH3) by DNA damage, which directly activated B⁃cell lymphoma, leukaemia⁃2⁃associated X protein (BAX), and B⁃cell lymphoma, leukaemia⁃2⁃associated K protein (BAK) and made them become homologous oligosaccharides. At this time, due to the penetration of outer mitochondrial members, mitochondrial intermembrane proteins are released in the cytoplasm and combine with *Apaf-1* to form procaspase-9-activating a heptameric protein complex called the apoptosome, which ultimately activates *caspase-3* and *caspase-7* cleaves the target protein, led to apoptotic breakdown (77). Moreover, in “type II” cells, such as hepatocytes, the mitochondrial amplifying loop activates the effector caspase and then releases SMAC to downregulate XIAP expression to mitigate its mediated caspase inhibition (77). Previous studies have shown that lycopene reduced carcinogenesis by inhibiting the phosphatidyl-inositol 3-kinase/serine–threonine kinase (PI3K-AKT) signaling pathway, similarly, lycopene induced apoptosis by downregulating B, cell CLL/lymphoma-2 (BCL-2), and upregulating BAX (79, 89). Previous records have shown that lycopene might increase the levels of Bax and E-cadherin and downregulate the levels of N-cadherin, phosphatidylinositol 3-kinase (p-PI3K), protein Kinase B (p-AKT), BCL-2, and phosphatidylinositol 3-kinases (PI3K)/AKT/mammalian target of rapamycin (p-m-TOR). The inactivation of PI3K/AKT/m-TOR signaling inhibits epithelial-to-mesenchymal transition apoptosis in oral cancer cells (13, 14). Moreover, lycopene could inhibit the proliferation of human breast cancer MCF-7 cells and promote their apoptosis by upregulating the gene expression of p53 protein (p53) and Bax (78).

It was concluded by Ozkan et al. (10) that that lycopene could play an anti-inflammatory and antioxidant role in ovarian cells by upregulating *Nrf2* or downregulating nuclear factor-kB ( *NF-kB* ) and *STAT3* signals, thus inhibiting the occurrence of spontaneous ovarian cancer in laying hens. Furthermore, lycopene also downregulated the mRNA expression of integrin β1 ( *ITGB1* ), integrin α5 ( *ITGA5* ), focal adhesion kinase ( *FAK* ), integrin-linked kinase ( *ILK* ), matrix metalloproteinase 9 ( *MMP9* ) and the expression of epithelial to mesenchymal transition (EMT) inhibits the activity of mitogen-activated protein kinase ( *MAPK* ) and reduced the mRNA expression level of the ovarian cancer biomarker CA125, thereby inhibited the proliferation of primary ovarian cancer cells and metastatic cells in mice (76). Cheng et al. (90) concluded that low-dose lycopene increased the expression level of 8-oxoguanine DNA glycosylase ( *OGG1* ), Nei-like DNA glycosylase ( *NEIL1* , *NEIL2* , *NEIL3* ), and connexin 43 (Cx43). Similarly, it also upregulated the expression level of scavenger receptor (SRB) protein SR-B1, thereby inhibiting lung cancer by improving gene stability and inhibiting smoke-induced oxidative stress. Lycopene inhibited the proliferation of ferret liver cancer and lung cancer cells induced by tobacco carcinogens by inhibiting the protein expression levels of lung α7 nicotinic acetylcholine receptor or *NF-kB* and cytochrome P450E1 (80). Additionally, it was concluded by Jhou et al. (91) that 2.5 μM lycopene could significantly downregulate the activities of NADPH oxidase 4 ( *NOX4* ), matrix metalloproteinase 2 ( *MMP-2* ) and matrix metalloproteinase 9 ( *MMP-9* ), and then inhibited the migration of human hepatic adenocarcinoma SK-Hep-1 cells. Moreover, inhibiting the migration of human umbilical vein endothelial cells and the activity of vascular endothelial growth factor are also two ways in which lycopene could effectively prevent cancer (92, 93).

In conclusion, Lycopene could induce mitochondrial apoptosis induced by the inactivation of the growth factor ( *FASR* , *VEGFR* , *IGF-1R* , and *PDGFR* ), mainly inducing BH3 dependent and independent activation of BAX and BAK, Ras/RAF/MAPK, and PI3K/AKT/PKB signaling pathways to achieve anticancer effects. When these pathway pathways are activated. Many related anti-cancer factors will be activated, thereby regulating the cell cycle, inflammation, apoptosis, metastasis, angiogenesis, and so on, ultimately achieving the purpose of anti-cancer.

### 3.4 Anti-inflammatory and immunomodulatory functions of lycopene

**Figure 6:**
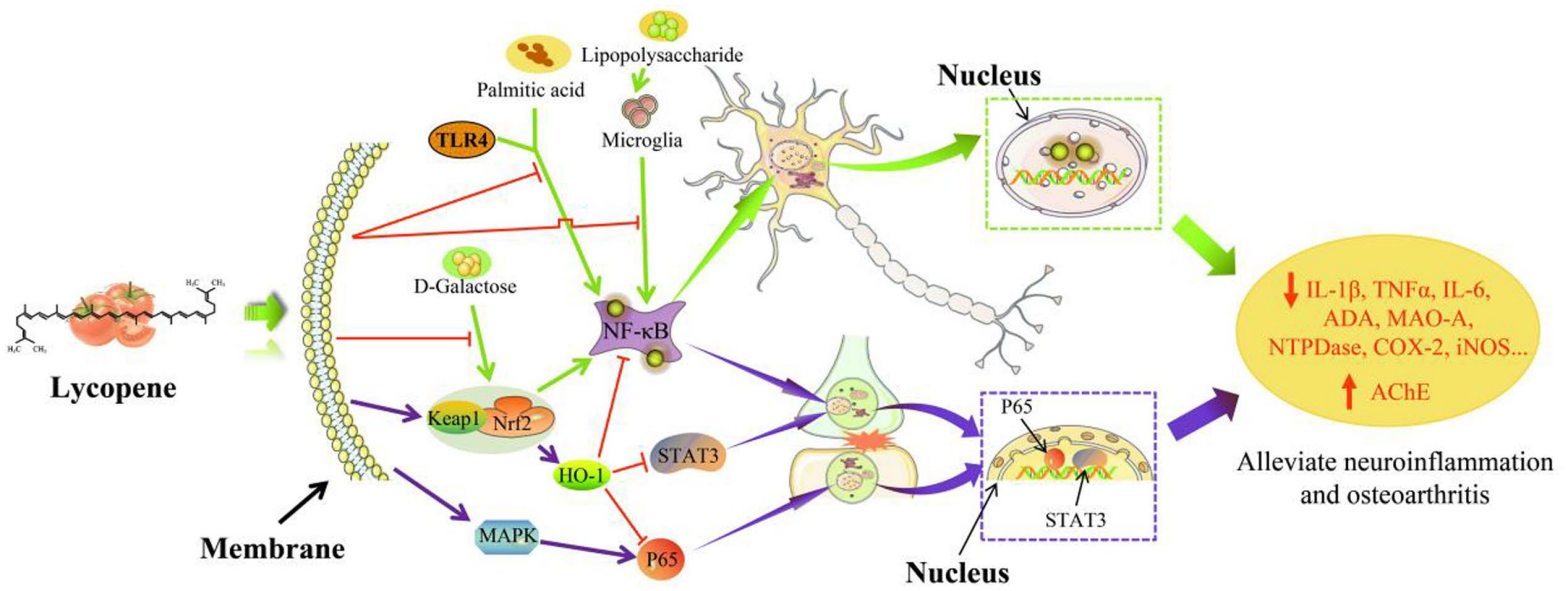
The main anti-inflammatory mechanism of lycopene (94–98).

The anti-inflammatory mechanism of lycopene was shown in Figure 6. Lycopene improved inflammation in the body by upregulating and downregulating many signaling channels and limited the transcription and expression of inflammatory mediator-related factors (34). As a key transcription factor involved in body inflammation and cellular immunity, *NF-kB* will participate in the transcription and synthesis of inflammatory cytokines and chemokines in acute inflammatory responses after activation and has powerful anti-apoptotic and endothelial anti-inflammatory effects. Therefore, it plays an important role in the body’s anti-inflammatory and immune regulation (99, 100). A previous study by Ugbaja et al. (101) indicated that lycopene reduced oxidative stress and downregulated the toll-like receptor-4 (TLR4) / NF- *ĸ* B-p65 axis in female Wistar rats, thereby mitigating the neuroinflammation caused by palmitic acid (PA). Furthermore, the reduction of lipopolysaccharide (LPS) induced neuroinflammation in male C57BL/6 J mice is affected by lycopene through the regulation of *MAPK* , *NF-κB* , and *Nrf2* signaling pathways (95). It was concluded by Zhao et al. (102) that lycopene mediated the Nrf2/NF- *ĸ* B pathway in CD-1 mice, which reduced neuroinflammation brought on by oxidative stress. Lycopene could activate the Nrf2/HO-1 signaling pathway, thereby inhibited the activation of the *NF* - *κB* and *STAT3* pathways caused by interleukin-1β (IL-1β), limited tumor necrosis factor-α (TNF-α), interleukin-6 (IL-6), cyclooxygenase-2 (COX-2), and inducible nitric oxide synthase ( *iNOS* ) gene transcription, and then reduced TNF-α, IL-6 and prostaglandin content, and slow down arthritis in mice (98). Moreover, lycopene also reduced arthritis by activating the Kelch-like epichlorohydrin-related protein-1 ( *Keap1* )-Nrf2 signaling pathway (97). In improving cellular inflammation, lycopene has been reported to stimulate *PPARγ* gene transcription by inhibiting *MAPK* and *NF* - *κB* signaling, on the other hand, lycopene also activated the Nrf2/HO-1 pathway, enhanced autophagy in liver macrophages, inhibited the expression of nucleotide-binding oligomerization domain-like receptor protein 3 ( *NLRP3* ), and reduced the secretion of IL-1β, IL-6, interleukin-8 (IL-8), and TNF-α ultimately achieves the purpose of improving macrophage inflammation (94, 96). Previous research has shown that the combination of quercetin and lycopene reduced the expression levels of modulatory metalloproteinase 7 ( *MMP7* ), MDA, MPO, and hydroxyproline, while the expression levels of SOD and GSH was elevated and has the potential to resolve inflammation induced by ochratoxin A ( *OTA* ) in rats (103). Notably, lycopene inhibited monocyte adhesion and migration induced by high mobility group protein box 1 (HMGB1) produced by necrotic cells and immune cells exposed to pro-inflammatory signals by decreasing the gene expression of cell adhesion molecules. Consequently, it reduced LPS-induced HMGB1 release and HMGB1-mediated secretion of TNF-α and secretory phospholipase A2, and the body finally exerts its anti-inflammatory function (104, 105).

**Figure 7:**
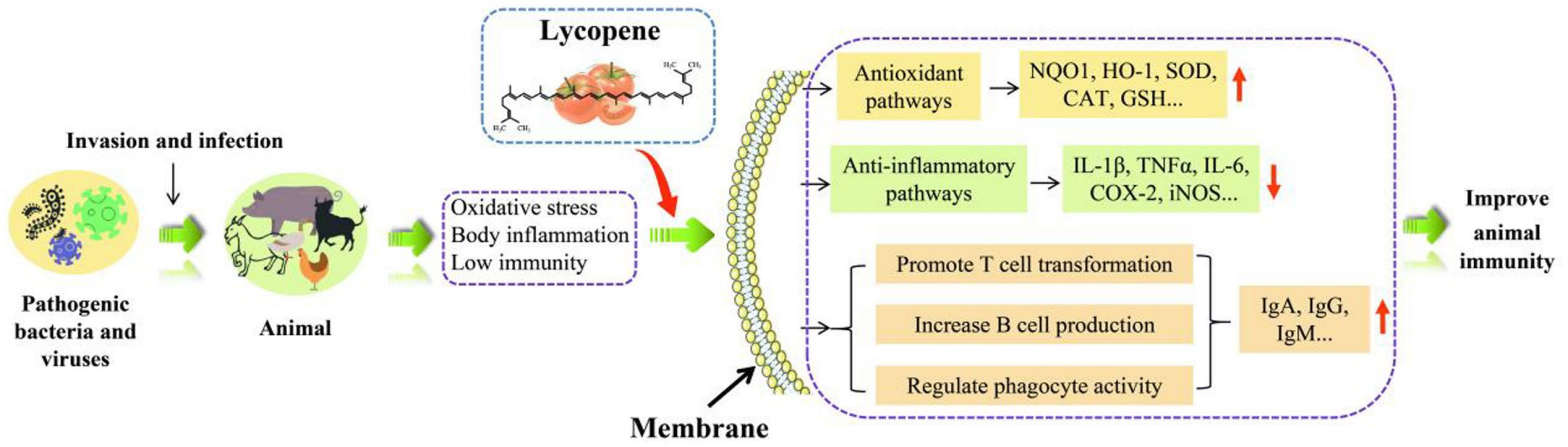
The main immunomodulatory mechanism of lycopene (16, 106–108).

In addition to inhibiting inflammation in the body, lycopene also has the function of enhancing cellular immunity and humoral immunity. The immunomodulatory of lycopene was shown in Figure 7. Furthermore, lycopene also has the function of protecting lymphocytes, promoting the transformation of T lymphocytes, increasing the CD4+/CD8+ ratio, and enhancing the activity of natural killer cells (109–111). It was concluded by Sarker et al. (112, 113) that lycopene reduced the damage aflatoxin B1 (AFB_1_) caused to broiler intestinal immune function by upregulating the production of claudin-1 (CLDN-1) mRNA and lowered the levels of the inflammatory cytokine interferon-γ (IFN-γ) and IL-1β. Similarly, Hidayat et al. (19) also obtained the same result. Lycopene might work by encouraging T-cell transition, raising the CD4+/CD8+ ratio, and concurrently lowering inflammatory cytokine levels, all of which would enhance rats’ immunity (106). Moreover, lycopene could also ensure the health of the sow placenta by reducing the expression level of placental inflammatory factors and improving immunoglobulin content, antioxidant capacity, placental lipid transport, and lipid metabolism (16). Other studies have found that lycopene could regulate the efficiency of IFN-γ and IL-2 production by mouse T lymphocytes or indirectly activate T cells (108). Furthermore, lycopene could increase the production of spleen B lymphocytes and serum immunoglobulin G content in mice, thereby improving the immune function of mice (107).

In conclusion, lycopene has potent anti-inflammatory and immunoregulatory properties. It could promote the development of B and T lymphocytes, stimulate the normal differentiation of T cells, along regulate the production and release of factors linked to inflammation.

## 4 Application of lycopene in poultry and ruminant production

As a new type of health additive and dietary supplement, lycopene has many functions in animal production, such as improving production performance, intestinal health, meat quality, and increasing animal reproduction rate, and has good application potential.

### 4.1 Application of lycopene in poultry production

Lycopene was widely used in poultry production because of its strong antioxidant, anti-inflammatory, and immune-regulating effects. In the current poultry research reports, lycopene was mainly used in poultry feed as a feed additive. It could be concluded from Table 2 that lycopene could reduce the feed conversion ratio (FCR), belly fat weight, and the levels of MDA, LDL-, TG, T-CHO, IFN-γ, IL-1β, ROS, and H_2_O_2_ in poultry. In addition, lycopene reduced the rate of drip loss, cooking loss, and pH drop in broilers. The incorporation of lycopene in poultry diets could improve the growth performance, feed intake, and meat quality of poultry. Moreover, lycopene could also improve the GSH-PX, GSH, GST, SOD, immune organ index, and HDL-C indicators of poultry. In gene expression, lycopene increased the mRNA expression levels of *Nrf2*, *ABCG5*, *ABCG8*, *Cludin-1*, *PGC1α*, *PPARα*, *RXRα*, *RARα*, and *RXRγ*. Furthermore, lycopene reduced the activity of acetyl-CoA carboxylase, sterol regulatory element binding protein 1 (SREBF1), fatty acid synthase, microsomal triglyceride transfer protein (MTP), Niemann-Pick C1Like1 ( *NPC1L1* ), cholesterol *O*-acyltransferase (ACAT) 2 ( *ACAT2* ), Which lycopene also reduced the mRNA expression levels of *SREBP1c*, *LXRα*, *ACLY*, *FABP1*, *FABP10* and *FATP4*. Lycopene increased the activity of intestinal digestive enzymes amylase, lipase, glutamine-cysteine ligase, mitochondrial glutathione, electron transport chain complex III, V, and manganese superoxide dismutase (MnSOD) in poultry. A previous study by Ayo et al. (116) indicated that lycopene could increase the concentration of plasma triiodothyronine concentration and the count of preovulatory follicles and white follicles. Furthermore, the temperature of the cloacal is reduced, thus increasing the reproductive rate of laying hens.

**Table 2:** Effect of supplement lycopene in feed in poultry.

Experimental animals	Optimal addition amount	Total experiment time	Effects on animal production (compared with the control group)	References
Japanese quail	300.00 mg/kg	28 d	↑ Growth performance and the levels of SOD. ↓ The levels of MDA and T-CHO in the muscle.	Amer et al. (68)
Male broilers	30.00 mg/kg	42 d	↑ The activity of Heme oxygenase 1, Nrf2, superoxide dismutase 2, and NAD(P)H quinone dehydrogenase 1.	Wang et al. (69)
Arbor Acres broilers	100.00 mg/kg	42 d	↑ The levels of GSH, GST, glutamine-cysteine ligase (GCL), CAT, and GSH-Px. ↓ The content of belly fat and the levels of LDL-C, ROS, MDA, TG, 8-OHdG, and T-CHO. ↓ The activity of SREBF1, acetyl-CoA carboxylase, and fatty acid synthase. ↓ The mRNA expression levels of *CYP2A6* and *CYP1A* genes.	Wan et al. (56)
Male broilers	100.00 mg/kg	42 d	↑ Growth performance, and the levels of HDL-C, CAT, and GSH-Px. ↓ FCR and the levels of T-CHO TG, LDL-C, and MDA. ↓ The activity of Alkaline phosphatase and alanine aminotransferase (ALT).	Mezbani et al. (114)
Arbor Acres broilers	200.00, 400.00 mg/kg	42 d	↑ Average daily gain (ADG), intestinal digestive enzyme amylase, and lipase activity. ↑ The concentration of mitochondrial glutathione (mGSH), GSH-Px, electron transport chain (ETC) complex III, V, and manganese superoxide dismutase (MnSOD), adenosine triphosphate. ↓ FCR. ↓ The levels of ROS, H_2_O_2,_ and mitochondrial swelling.	Sarker et al. (112, 113)
Laying hens	200.00 mg/kg	84 d	↑ The mRNA expression levels of *ABCG5* and *ABCG8* genes. ↓The mRNA expression levels of *NPC1L1*, *ACAT2*, *MTP*, *SREBP1c*, *ACLY*, and *LXRα* genes.	Orhan et al. (115)
Xinghua breeding hens	40.00, 80.00 mg/kg	42 d	↑ The mRNA expression levels of *PGC1α*, *PPARα*, *PPARγ*, *RXRα*, *RXRγ*, and *RARα* genes. ↓ The mRNA expression levels of *FABP1*, *FABP10*, and *FATP4* genes.	Tian et al. (55)
Laying hens	30.00 mg/kg	35 d	↑ Plasma triiodothyronine concentration, preovulatory follicles count, white follicles count, and egg production performance. ↓ Cloacal temperature.	Ayo et al. (116)
Japanese quail	100.00 mg/kg	49 d	↑ The levels of GSH-PX and SOD. ↓ The levels of serum T-CHO, TG, LDL-C, aspartate aminotransferase (AST), ALT, and MDA.	Abbas et al. (117)
Broilers	200.00 mg/kg	42 d	↑ ADG, immune organ index, and meat quality. ↓ The rate of drip loss, cooking loss, and pH drop.	Fu et al. (118)
Hyline brown hens	100.00 ng/mL	72 h	↓ D-galactose induced mitochondrial damage in living granulosa cells through the Nrf2/HO-1 pathway.	Liu et al. (119)

↑, increase; ↓, decrease; d, days; h, hours.

### 4.2 Application of lycopene in ruminant production

Lycopene has been widely used in animal production because of its special beneficial functions to animals. There are surprisingly few reported lycopene-related outcomes in ruminant production, according to the most recent published reports. It could be concluded from Table 3 that supplementing lycopene to sheep and goat diets could improve T-AOC, CAT, GSH-Px, SOD, vitamin E, production performance, slaughter performance, and meat quality. Additionally, it was concluded by Fallah et al. (15) that lycopene could enhance immunological markers, sex hormones, and milk quality, which promote the breast development of ewes. The incorporation of lycopene in diets reduced the levels of T-CHO, TG, LDL-C, and MDA, which reduced FCR, Muscle L* value, the content of muscle fat, lipid oxidation, and protein oxidation (including thiobarbituric acid-reactive substance and carbony l) in sheep and goats. In addition, in an experiment on the effect of lycopene on ram semen, it was concluded that supplementing lycopene improved the individual motility of goat sperm and reduced sperm mortality and abnormal sperm content. The application of lycopene in cattle diets production is rarely reported. Lycopene improved the milk quality, embryo development, and quantity of cattle, and reduced ROS levels and the expression of genes such as *NF-kB, iNOS, BCL-2* and *COX2*.

**Table 3:** Effect of supplement lycopene in ruminant production.

Experimental animals	Optimal addition amount	Total experiment time	Effects on animal production (compared with the control group)	References
Bamei sheep	200.00 mg/kg	120 d	↑ ADG and meat quality. ↑ The levels of plasma antioxidant vitamin E, T-AOC, CAT, GSH-Px, and SOD. ↓ The levels of T-CHO, TG, LDL-C, lipid oxidation, muscle fat, therosclerosis index in plasma, and MDA in plasma and liver.	Jiang et al. (120, 121)
Hu sheep	200.00 mg/kg	90 d	↑ The levels of T-AOC, SOD、CAT, GSH-Px, Muscle a* value, vitamin A/ E, and FCR. ↓ The levels of drip loss, lipid, protein oxidation, thiobarbituric acid reactive substance (TBARS), and carbonyl.	Wang et al. (122) and Xu et al. (123)
Hu sheep	200.00 mg/kg	110 d	↑ ADG，chest width，body length, height at withers, chest circumferencepre-slaughter live weight, Longissimus dorsi muscle weight, kidney weight, GR value, and leather weight. ↑ The activities of serum catalase and glutathione peroxidase. ↓ MDA.	Qu et al. (124)
Single-bearing Lori-Bakhtiari ewes	100.00 mg/day	21 d	↑ Udder volume and the quantity of colostrum. ↑ The levels of protein in colostrum, circulating glucose, prolactin, estradiol, lymphocyte cells, and circulating IgG. ↓ The levels of urea, progesterone, and the ratio of progesterone to estradiol.	Fallah et al. (15)
Turkish Awassi ram semen	0.30%	95 d	↑ The levels of sperm’s individual motility. ↓ The levels of sperm mortality, abnormal sperm, and sperm acrosomal damage.	Al-Sarray et al. (125)
Merino Ram Semen	0.50, 2.00 mM	72 h	↑ Mitochondrial activity rate, sperm motility rate, and GSH levels. ↓ The levels of lipid peroxidation.	Akalin et al. (126)
Cashmere goat semen	1.00 mg/ mL+ 5.00 μg/mL (Lycopene+Alpha-lipoic acid)	21 d	↑ The levels of sperm motility, acrosome integrity, membrane integrity, pregnancy rates, mitochondrial activity, SOD, CAT, and GSH-Px.	Ren et al. (127)
Italian Friesian dairy cows	1.27 g/day/cow	21 d	↑ The levels of energy corrected milk, milk fat, and T-AOC. ↓ The levels of TBARS and log10 somatic cell count.	Garavaglia et al. (128)
Bovine cumulus-oocyte Bovine embryo	0.20 μM	22 h	↑ The number of blastocysts and BCL2. ↓ The levels of ROS and apoptosisc. ↓ The mRNA expression levels of NF-kB, COX2, iNOS, BAX genes.	Chowdhury et al. (129)
Bovine cumulus-oocyte Bovine embryo	0.20 μM	8 d	↑ The total cell, trophectoderm, inner cell mass numbers, and nuclear maturation rate. ↓ The oocyte ROS production.	Residiwati et al. (130)

↑, increase; ↓, decrease.

In conclusion, we have reviewed many existing literature reports and found that the incorporation of lycopene in diets of poultry and ruminant feeding could improve growth performance, meat quality, and reproductive performance, but the optimal amount of lycopene has a large span. Existing reports have found that the optimal amount of lycopene added to poultry feed ranges from 30.00 mg/kg to 400.00 mg/kg. Additionally, in the feeding of ruminants, the amount of lycopene added to the feed should not exceed 200.00 mg/kg. From the data obtained, it can be concluded that the study of the optimal addition amount of lycopene in poultry and ruminant feed is still a hot topic, which needs to be further verified in the future.

## 5 Conclusion

Lycopene is a new type of healthy feed additive and has been gradually applied in animal production because of its natural, pollution-free, non-toxic, and side effects, and its physiological functions such as anti-inflammatory, antioxidant, and immune regulation. Lycopene could be regulated through many mechanisms to improve the antioxidant, immunity, meat quality, and reproductive rate of animals. However, the existing research reports found that lycopene is mainly used in poultry research in animal production research, while research reports on ruminants are relatively rare and many studies are still in their infancy.

In conclusion, the current research on lycopene in animal production is still relatively limited, not systematic and in-depth enough, and the amount of lycopene added to different types of animal feed is not clear enough, and its mechanism of action and targets need to be further explored. Therefore, research on the application of lycopene in different physiological conditions and growth stages of animals can be strengthened in the future. Notably, explore the appropriate addition amount of lycopene in feed and reveal its specific mechanism of action. This is of great significance to promote the development of lycopene feed and the ecological and healthy breeding of livestock.

## Author contributions

YL: Data curation, Writing – original draft, Writing – review & editing. SP: Investigation, Methodology, Visualization, Writing – review & editing. SL: Data curation, Writing – review & editing. XN: Formal analysis, Supervision, Validation, Writing – review & editing. ST: Data curation, Investigation, Methodology, Writing – review & editing. NT: Conceptualization, Methodology, Writing – review & editing. YH: Investigation, Project administration, Writing – review & editing. PP: Project administration, Resources, Writing – review & editing.
